# Overcoming Immune Resistance in Prostate Cancer: Challenges and Advances

**DOI:** 10.3390/cancers13194757

**Published:** 2021-09-23

**Authors:** Miyad Movassaghi, Rainjade Chung, Christopher B. Anderson, Mark Stein, Yvonne Saenger, Izak Faiena

**Affiliations:** 1 Department of Urology, Columbia University Irving Medical Center, New York, NY 10032, USA; rc3178@cumc.columbia.edu (R.C.); cba2125@cumc.columbia.edu (C.B.A.); 2 Department of Medicine, Division of Medical Oncology, Columbia University Irving Medical Center, New York, NY 10032, USA; mns2146@cumc.columbia.edu (M.S.); yms4@cumc.columbia.edu (Y.S.)

**Keywords:** immunotherapy, metastatic castration resistant prostate cancer, tumor microenvironment, immune resistance, combination therapies, immune checkpoint inhibitors

## Abstract

**Simple Summary:**

Immunotherapy has changed the landscape of treatment modalities available for many different types of malignancies. However, the factors that influence the success of immunotherapeutics have not been as clearly seen in advanced prostate cancer, likely due to immunosuppressive factors that exist within the prostate cancer tumor microenvironment. While there have been many immunotherapeutics used for prostate cancer, the majority have targeted a single immunosuppressive mechanism resulting in limited clinical efficacy. More recent research centered on elucidating the key mechanisms of immune resistance in the prostate tumor microenvironment has led to the discovery of a range of new treatment targets. With that in mind, many clinical trials have now set out to evaluate combination immunotherapeutic strategies in patients with advanced prostate cancer, in the hopes of circumventing the immunosuppressive mechanisms.

**Abstract:**

The use of immunotherapy has become a critical treatment modality in many advanced cancers. However, immunotherapy in prostate cancer has not been met with similar success. Multiple interrelated mechanisms, such as low tumor mutational burden, immunosuppressive cells, and impaired cellular immunity, appear to subvert the immune system, creating an immunosuppressive tumor microenvironment and leading to lower treatment efficacy in advanced prostate cancer. The lethality of metastatic castrate-resistant prostate cancer is driven by the lack of therapeutic regimens capable of generating durable responses. Multiple strategies are currently being tested to overcome immune resistance including combining various classes of treatment modalities. Several completed and ongoing trials have shown that combining vaccines or checkpoint inhibitors with hormonal therapy, radiotherapy, antibody–drug conjugates, chimeric antigen receptor T cell therapy, or chemotherapy may enhance immune responses and induce long-lasting clinical responses without significant toxicity. Here, we review the current state of immunotherapy for prostate cancer, as well as tumor-specific mechanisms underlying therapeutic resistance, with a comprehensive look at the current preclinical and clinical immunotherapeutic strategies aimed at overcoming the immunosuppressive tumor microenvironment and impaired cellular immunity that have largely limited the utility of immunotherapy in advanced prostate cancer.

## 1. Introduction

Androgens have a key role in the pathogenesis of prostate cancer (PCa), and treatment modalities altering androgen receptor signaling pathways are the standard of care for advanced and disseminated disease. However, despite the initial effectiveness of androgen deprivation therapy (ADT), resistance to therapy occurs in approximately 30–50% of patients, resulting in castration-resistant prostate cancer (CRPC) for which there are very limited, generally not curative, systemic treatment options [1,2].

Utilizing the immune system to treat cancer has been a revolutionary development and has quickly become the standard treatment for many cancer types, superseding other targeted and systemic therapies [3]. By targeting cancer cells and avoiding the toxicities of chemotherapy and radiation, immunotherapy offers a less toxic, yet, in many types of cancers, highly efficacious alternative [4]. With regard to PCa, the interaction between prostatic epithelial cells and the immune and non-immune cells that make up the tumor microenvironment (TME) have been shown to have an important role in the complex changes that occur and ultimately result in disease progression, development of resistant metastases, and the overall resistance to both conventional and experimental therapies [1,5].

To date, however, patients with advanced PCa have not yet benefited to the same extent as those with more “immunologically hot” or “responsive” tumors such as melanoma, lung cancer, renal cell carcinoma or urothelial carcinoma [3,6,7]. In fact, PCa has been classified as a “cold tumor” with minimal response to immune-related treatment modalities [8,9,10]. Low tumor-associated antigen expression, decreased major histocompatibility complex (MHC) presentation of tumor antigens, tumor suppressor and DNA repair enzyme defects, and poor immune-modulating signaling are some key processes that have a role in this complex tumor environment, altering the overall anti-tumor response [8,11]. Efforts have been made to target these immune evasion mechanisms in CRPC. Currently ongoing preclinical and clinical trials are reporting encouraging results on combination-based therapies. However, other than pembrolizumab, which was approved for advanced solid tumors from the US Food and Drug Administration (FDA) in 2017 for high microsatellite instability (MSI) and in 2020 for high tumor mutational burden (TMB), sipuleucel-T, an immunotherapy based on the infusion of antigen presenting cells (APCs) which has demonstrated improvements in survival without a significant response rate, remains the only FDA-approved immunotherapy for mCRPC [12,13].

**Figure 1:**
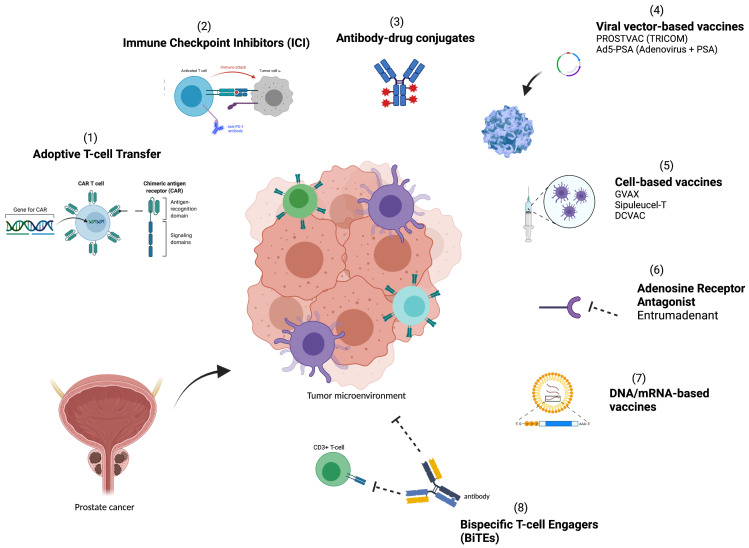
Immunotherapeutic categories designed to overcome immune resistance mechanisms in the tumor microenvironment of prostate cancer: (**1**) adoptive cell transfer, (**2**) Immune checkpoint inhibitors, (**3**) antibody–drug conjugates, (**4**) viral vector-based vaccines, (**5**) cell-based vaccines, (**6**) Adenosine receptor antagonists; (**7**) DNA/mRNA- based vaccines, and (**8**) Bispecific T-cell engagers.

A greater understanding of the TME and methods for utilizing the host immune system to halt and eliminate tumor growth is needed to improve therapies. In this review, we set out to identify and explore key immune resistance mechanisms that lead to treatment failure in the immunosuppressive TME of PCa, and focus on therapeutic strategies (Figure 1) and approaches that target the immunosuppressive TME and seek to overcome these resistance mechanisms.

## 2. Immune Resistance Mechanisms in Prostate Cancer

In most solid tumors, effective immune responses within the TME rely on an increased infiltration and activation of immune cells, increased mutational burden within the cancer cells, expression of these tumor antigens on the cell surface, functional immune signaling pathways, and appropriate tumor suppressor functions [14,15]. Mechanisms that bypass these coordinated cellular functions ultimately result in immune evasion and subsequent malignant disease progression, and are thought to be critical factors in limiting the response to immune therapies [16].

The immunologic classification of tumors as “hot” or “cold” is determined by parameters that include the presence of specific tumor-infiltrating lymphocytes (TILs) such as CD4^+^ and CD8^+^ T cells, high TMB, as well as expression and recognition of neoantigens via MHC molecules by APCs. “Hot” tumors trigger an antitumor immune response based on these factors [9]. Conversely, “cold” tumors have been shown to have low TILs, impaired APC activation and presence of immune-suppressive cell types. PCa falls within the category of a “cold” tumor [9,17].

The mechanisms through which PCa evades the immune system, maintains the “cold” TME and mediates immunosuppression continues to be elucidated. In order to best overcome the resistance to immunotherapies in the setting of advanced PCa, it is critical to understand the mechanisms that lead to immune resistance in the context of the cellular components of the TME that potentiate immunosuppression and the molecules and genetic pathways that facilitate continued tumor growth [9]. Some of these key immune resistance mechanisms that will be explored include altered MHC molecule expression, decreased TMB, loss of tumor suppressor proteins, abberant androgen receptor signaling as well as increased infiltration of immune-suppressive cell types (i.e., T-regulatory cells, myeloid-derived suppressor cells) in the TME [9,17,18].

### 2.1. Role of Myeloid-Derived Suppressor Cells in the TME

In the setting of PCa, it has been shown that tumor development and progression is associated with a local inflammatory state, leading to the accumulation of an immune-suppressive population of Myeloid-derived suppressor cells (MDSC) within the TME [19]. MDSCs make up a group of immature myeloid cells (IMCs) that have been shown to exhibit strong immuno-suppressive functions of T and natural killer (NK) cells. Under normal conditions, the MDSCs differentiate into macrophages, granulocytes or tissue-resident dendritic cells. During an inflammatory response, these immature myeloid cells become activated to monocytes and neutrophils [20].

Over time, prostate tumor cells accumulate genetic alterations that allow them to move from the primary tumor site and metastasize to different anatomic sites. Bone, being a major metastatic site for advanced PCa in humans, has been found to have large populations of MDSCs in animal models [21,22]. Hossain et al. found a significantly increased percentage of MDSC in the blood of both mCRPC and localized tumor groups compared to the controls [23]. MDSCs were also found to be significantly increased in the circulation of mCRPC patients, in both untreated and docetaxel-treated groups compared to the controls, with significantly inhibited CD4^+^ T-cell proliferation seen in both groups [24]. Furthermore, previous studies have also demonstrated an upregulation in the production of chemokines that promote the recruitment of MDSCs to the PCa TME, with a remarkable decreased expression of small molecules responsible for the recruitment of cytotoxic T lymphocytes (CTL) [25]. This provides further evidence that the PCa TME, in part, promotes the presence of these immune suppressor cells leading to a diminished anti-tumor immune response that may otherwise help control progression of the disease. 

### 2.2. Altered Major Histocompatibility Complex Class I Expression

Major Histocompatibility Complex (MHC) Class I proteins are typically found on the cell surface and have an important anti-tumor role presenting tumor antigens to CTLs resulting in an immunostimulatory signaling cascade leading to T cell activation and target cell destruction [8,26]. Loss of MHC I is one mechanism of immune evasion [26] that has been demonstrated in metastatic PCa cell lines and clinical specimens. Loss of MHC I occurs through defective synthesis and transport of MHC molecules, processing of tumor antigens, and loss of critical proteins required for cell-surface expression [27]. 

### 2.3. Low Tumor Mutational Burden

Neoantigen expression on the cell surface of tumor cells via MHC Class I/Class II molecules and activation of APCs and CTLs is essential in generating an anti-tumor immune response leading to tumor cell apoptosis [28]. TMB is a quantitative measure of the total number of mutations per coding region. Because some cancers result in a higher TMB, there is a subsequent increase in tumor neoantigen expression and thus, anti-tumor immune response [28]. PCa, however, has low somatic TMB and thus, decreased neoantigen expression compared to other tumor-types [8]. In fact, one study found mean somatic mutational rates at a frequency of 0.9 per megabase, approximately 10 times lower than reported for melanoma [29]. This highlights a potential lack of T-cell co-stimulation and activation in the PCa TME, which prohibits the generation of a powerful adaptive immune response following antigen presentation; a key step in immunotherapy effectiveness [8].

### 2.4. Interferon Pathway

Interferons (INF1) are a group of immunostimulatory cytokines released in response to cellular detection of invading pathogens [8]. Activation and expression of *INF1* gene has also been shown to be crucial in mounting an efficient anti-tumor immune response through the release of cytokines that ultimately lead to an increase in the expression of immune costimulatory molecules, activation of adaptive immune cells, and an increase in TIL killing [30].

Interferon-gamma, INF-γ, a potent cytokine known to modulate tumor immunity and tumoricidal effects, has been shown to be highly elevated in patients with PCa after radiation [31]. Kundu et al. found that IFNγ can induce epithelial-to-mesenchymal transition in PCa cells leading to the downstream activation and expression of IFN-stimulated genes, PCa cell death and tumor regression [31,32]. Further animal models have also demonstrated that loss of tumor-intrinsic type I IFN can occur in proliferating PCa cells in bone and that the loss suppresses anti-tumor and therapeutic responses, in addition to promoting bone PCa cell activation and cancer progression [33]. These all highlight the importance of INF1 and their role in an anti-tumor immune response. 

### 2.5. Loss of PTEN

Tumor suppressor proteins have an important role in immune cell maturation and activation. Within the TME, loss of these proteins can limit several immune response pathways that lead to a pro-tumorigenic state. *PTEN* has been shown to antagonize signaling pathways that lead to tumor growth while modulating the activation of INF1 and NK-κB pathways [34]. In PCa, mutations or deletions in *PTEN* have been found in up to 25% of primary cancers after radical prostatectomy and as many as 70% of mCRPCs. Similarly, *PTEN*-deficient prostate cancers have been shown to have higher regulatory T cell densities, suggesting an expansion of immunosuppressive factors in the TME [34]. In other cancers, such as melanoma, *PTEN*-silenced mouse models demonstrate a significantly reduced therapeutic activity of tumor-specific TILs compared with those with an intact *PTEN* gene [35]. Although these findings need to be validated in PCa, the data provide evidence for a possible mechanistic role of *PTEN* in altering the immune milieu and response to immunotherapy in the PCa TME. 

### 2.6. Androgen Receptor Signaling

The nuclear androgen receptor (AR) transcription factor has an important role in maintaining optimal function and physiology of the human prostate. The binding of dihydrotestosterone (DHT) to the AR triggers a cascade of pathways mediating this physiology. ADT works to limit signaling pathways critical to AR activation [36].

More recent evidence has shown that the use of ADT and androgen withdrawal can alter the PCa TME. In the short term, androgen deprivation either by surgical castration or anti-androgen therapy targeting the AR signaling pathway has been reported to result in an increase in the number of TILs, and decrease in the number of Tregs supporting an antitumor response to ADT [37]. PCa mouse models have demonstrated that ADT initially induces a pro-inflammatory infiltrate, but that this ultimately decreases with the development of castration resistance and induction of more immune tolerance to PCa antigens [38]. The initial infiltration of CD8^+^ T cells seen in the TME of men with high-risk localized PCa who received ADT plus a cell-based vaccine (Cy/GVAX) in the neoadjuvant setting is initially increased with the use of ADT. However, a proportional increase in FoxP3^+^ T regulatory cells (Tregs) was simultaneously seen, [39] suggesting that attempts to remove these Treg cells may be essential in stimulating an anti-tumor immune response following AR antagonist therapy.

## 3. Therapies Targeting Immune Resistance

### 3.1. Immune Checkpoint Inhibitors

Early Phase Studies

The development of immune checkpoint inhibitors (ICIs) has revolutionized oncologic immunotherapeutics over the last decade. Normally, checkpoint proteins and their corresponding receptors keep immune responses in check. Antibodies that inhibit the checkpoint inhibitors, programmed death ligand 1 (PD1/PD-L1) and cytotoxic T lymphocyte-associated protein 4 (CTLA-4), have demonstrated long-term survival benefits and antitumor effects in malignancies such as renal cell carcinoma, melanoma, non-small cell lung cancer, and urothelial carcinoma [40,41,42]. 

There has been conflicting evidence on the role of PD-L1 expression in the PCa immune response. Some studies have surprisingly demonstrated that low levels of PD-L1 expression is associated with PCa progression [43,44]. This may help explain why anti-PD-L1 monotherapies have had limited success in mCRPC. For example, PD-L1 expression was rarely observed in specimens of patients with primary PCa, especially in the context of PTEN loss, whereas PD-L1 expression was increased in response to proinflammatory signals in vitro [44]. Interestingly, in the neoadjuvant setting, one study reported downregulation of PD-L1 expression in radical prostatectomy tumor specimens in patients previously treated with abiraterone and prednisone [43].

On the other end, there have been reports suggesting increased PD-L1 expressed in mCRPC [45]. One study reported upregulation of PD-L1 expression in the PCa tissue and circulating dendritic cells in enzalutamide-resistant tissues in both patients and mouse models [46]. This observation suggests that resistance to enzalutamide may be associated with increased PD-L1 expression on the target immune and cancer cells, rather than a through an AR pathway. These variations in PD-L1 expression in PCa tumors suggest that levels of immune checkpoint molecule expression may vary at different stages of PCa progression and according to previous therapies received [45].

Most ICI monotherapies have shown limited survival benefit in patients with mCRPC, with the exception of pembrolizumab (PD-1 inhibitor), which is FDA-approved for microsatellite instability (MSI) high status, DNA mismatch repair (MMR) enzyme deficiency, or high tumor burden [47,48,49]. In the phase II KEYNOTE-199 study, pembrolizumab monotherapy demonstrated objective response rates between 3–5% in patients with mCRPC previously treated with docetaxel, as well as durable antitumor activity among patients with mCRPC resistant to enzalutamide [47]. Thus, the community has shifted towards studying combination therapies of two different ICI or combining ICI with other treatment modalities sequentially or in parallel.

The thought behind combinatorial strategies is to turn PCa from an immunologically “cold” cancer, to a more “hot” TME [44]. Data from the ongoing CHECKMATE-650 phase II trial, using the combination of anti-PD-1 (nivolumab) and anti-CTLA-4 (ipilimumab) therapy in patients with asymptomatic or minimally symptomatic mCRPC, some of whom had previously received cabazitaxel, reported an objective response of 26% in the chemotherapy-naive patients [50]. Determining the balance between efficacy and toxicity is critical when combining therapies. In the CHECKMATE-650 trial, treatment-related toxicity was significant, and only 40–50% of the patients completed all four treatment cycles. 

Apart from radiation therapy, the combination of other therapies with ICI is currently being evaluated and underway. The KEYNOTE-365 is a non-randomized phase Ib/II umbrella trial of patients with mCRPC who progressed on chemotherapy and hormonal therapy to evaluate the efficacy of pembrolizumab and olaparib (PARP inhibitor), pembrolizumab and docetaxel/prednisone, and pembrolizumab and enzalutamide. The objective response rate of chemotherapy-naïve, abiraterone-treated mCRPC patients receiving the combination of enzalutamide and pembrolizumab was shown to be 20% (NCT02861573) [51,52]. COSMIC-021, a phase Ib trial, is studying the combination of cabozantinib (tyrosine kinase inhibitor) and atezolizumab (PD-L/PD-L1 inhibitor) in mCRPC [53].

Late Phase Studies

More recently, a long-term survival advantage was reported in the phase III trial of iplilimumab versus placebo after radiotherapy in docetaxel-treated mCRPC patients [54]. However, another phase 3 trial (CA184-095) looking at ipilimumab in docetaxel-naïve mCRPC patients without any prior radiation showed no OS benefit, despite a significant improvement in progression-free survival (PFS) [6]. One thought is that the use of radiation and/or chemotherapy prior to ICI use can lead to improved survival advantages by converting the “cold” TME into a “hot” immunologically active TME. In preclinical studies, significantly increased survival advantages have been seen in murine CRPC models treated with radiation with either combination PD-1 or PD-L1 therapy compared to ICI alone [55]. In addition to direct killing of tumor cells and increased mutations within tumor-derived peptides, Philippou et al. demonstrated increased immune infiltration in the TME of mice with mCRPC, along with upregulated PD-1/PD-L1 expression and CD8^+^ T cell infiltration after radiotherapy treatment [56].

### 3.2. Cellular Therapies—Chimeric Antigen Receptor T Cells

Pre-clinical studies have focused on targeted therapies against specific cancer stem cells. Cancer stem cells are a subpopulation of cancer cells with self-renewing capabilities, which are distinguished by the types of proteins expressed on their cell surface. Common cell-surface markers used in the identification of PCa stem cells include CD44, CD133, and epithelial cell adhesion molecule (EpCaM) [57]. 

Cancer stem cell–targeting immunotherapy has recently been attempted in PCa with chimeric antigen receptor (CAR) T cells. CAR T cells are cell-based vaccines that are a burgeoning new area of interest in immunotherapy. The precisely ex vivo engineered receptors allow the T cells to recognize and bind to specific antigens or proteins on tumor cells. Once created, the CAR T cells are engineered to express a synthetic receptor that has high affinity for specific tumor cells targeting tumor-associated antigens (TAAs) in a non-HLA complex-restricted manner [58], and are then expanded and then infused into the patient to target and kill cells carrying the specific antigen.

PCa has several overexpressed cell-surface tumor antigens, such as EpCAM, prostate stem cell antigen (PSCA) and prostate specific membrane antigen (PSMA). In one preclinical model for metastatic PCa, CAR T cells were engineered against EpCaM-expressing cancer stem cells in a murine PCa model that demonstrated inhibited tumor growth and prolonged mouse survival [59]. A preclinical model showed that PSMA-directed CAR T cells combined with docetaxel induced PCa tumor regression in a xenograft model [60]. There are several promising ongoing trials using CAR T cells to target antigens expressed in PCa including PSCA (NCT02744287), PSMA (NCT01140373), EpCAM (NCT03013712), and NY-ESO-1 (NCT03159585). 

### 3.3. Vaccines

Vaccination has been an important strategy in addressing anti-cancer immunity. The major categories of anti-tumor vaccines include dendritic cell-based, viral vector-based, cell-based, peptide-based, and DNA/mRNA-based. As with other therapies in development, the use of vaccines in PCa requires a deep understanding of the key immune mechanisms in the TME, PCa mutational load, and the expression profile of PCa-specific tumor antigens [58].

#### 3.3.1. Dendritic and Other Cell-Based Vaccines

##### Sipuleucel-T

Early Phase Studies

The Sipuleucel-T vaccine involves the ex vivo expansion and activation of patient-derived peripheral blood mononuclear cells (PBMCs) with a recombinant prostatic acid phosphatase fusion protein [61,62]. The basis for this vaccine came from early mouse studies that showed dendritic cells could be removed, activated ex vivo with tumor-associated antigens, and replaced in vivo [63]. Sipuleucel-T has been investigated earlier in the course of PCa. A phase II trial assessed the use of neoadjuvant Sipuleucel-T in men who were planning to undergo radical prostatectomy, but did not observe any tumor downstaging [64]. There have also been a number of studies looking at its use in nonmetastatic biochemical recurrent (BCR) disease. One phase II study of Sipuleucel-T in 13/18 patients with BCR observed an increase in PSA doubling times (PSADT), but no decrease in absolute PSA levels [65]. Another phase II trial randomly administered Sipuleucel-T to patients with BCR after radical prostatectomy while on ADT. Although they also saw an increase in PSADTs, they did not find any difference in the primary endpoint of time to develop biochemical failure following ADT [66]. Therefore, current evidence does not support Sipuleucel-T use in patients with BCR after primary treatment, advanced disease with visceral mets, or in men with non-metastatic CRPC.

Late Phase Studies

Based on the phase III IMPACT trial [61], Sipuleucel-T was the first FDA approved autologous cellular therapeutic vaccine for patients with mCRPC with no visceral metastasis and little to no symptoms. In the trial, CRPC patients receiving the treatment had a median survival 4.1 months longer than placebo-treated patients, although the time to disease progression was not significantly different [61]. However, greater than 50% of patients in the placebo group with disease progression crossed over to receive the vaccine, and these patients had significantly longer overall survival (OS) times compared to the group that did not cross over (20 months vs. 9.8 months). Thus, the absolute survival benefit of Sipuleucel-T may be greater than initially observed.

##### GVAX Prostate

Early Phase Studies

GVAX is a GM-CSF-secreting tumor cell vaccine made from irradiating tumor antigens from the PCa hormone-sensitive cell line, LNCaP, and the hormone refractory cell line, PC-3 [67,68]. An initial phase I/II study where patients with metastatic hormone-refractory PCa were treated with GVAC saw a statistically significant decrease in PSA velocity and a trend towards a dose-dependent survival benefit in patients receiving the vaccine [68].

Late Phase Studies

This ultimately led to two phase III trials (VITAL-1 and VITAL-2) that were both terminated early based on the results of an unplanned futility assessment, where the combination of GVAX and docetaxel resulted in an increased mortality rate compared to the chemotherapy monotherapy group alone (NCT00133224). Although the use of GVAX in the mCRPC setting has been limited to date, the use GVAX in combination with cyclophosphamide followed by degarelix vs. degarelix alone is being investivated in the neoadjuvant setting in high risk localized PCa [39].

##### DCVAC/PCa

Early Phase Studies

DCVAC/PCa is a dendritic cell vaccine formed by in vitro activation of autologous dendritic cells with the LNCaP killed PCa cell line, which has shown conflicting results. One phase I/II trial observed a 6–7 months OS advantage for mCRPC patients receiving DCVAC/PCa and docetaxel/prednisone [69]. A subsequent phase II randomized trial of DCVAC with docetaxel compared to docetaxel alone did not observe a survival benefit in mCRPC [70]. 

Late Phase Studies

The phase III VIABLE trial results, presented at the 30th annual meeting of the Society for Immunotherapy of cancer, was a randomized, double-blinded, placebo-controlled trial, where DCVAC/PCa in combination with docetaxel and prednisone was compared to docetaxel/prednisone alone in mCRPC (NCT02111577). The preliminary report did not demonstrate a survival benefit in those receiving DCVAC/PCA, with OS times of 23.9 compared to 24.3 (results available on Clinicaltrial.gov NCT02111577).

#### 3.3.2. Viral Vector-Based Vaccines

##### PSA-TRICOM

Prostvac is a viral vector-based vaccine created by inserting a recombinant plasmid with a PSA transgene into a poxvirus along with plasmids coding for three viral T-cell costimulatory molecules (TRICOM). Arlen et al. found that the addition of TRICOM (LFA-1, ICAM, and B7-1) had a dramatic synergistic effect on catalyzing a targeted immune response compared to constructs without co-stimulatory molecules or carrying just one or two molecules [71]. Prostvac utilizes two different recombinant pox virus-based vectors (vaccinia and fowlpox viruses) to generate a T-cell response in PCa patients [72]. Patients first receive a vaccinia-based priming vaccine (Prostvac-V) and then receive monthly poxvirus-based vaccines as boosters (Prostvac-F). The effect of Prostvac in combination with GM-CSF was studied in a placebo-controlled phase II study in men with minimally symptomatic mCRPC who had not had prior chemotherapy. Patients who received Prostvac and GM-CSF had a 44% reduction in mortality [73]. However, the subsequent phase III PROSPECT trial that randomly assigned patients with mCRPC to received Prostvac with adjuvant GM-CSF or placebo showed no difference in overall survival [74].

##### Adenovirus/PSA

Research on adenovirus-based vaccines has been underway for decades, and highlighted by the recent use of the Johnson & Johnson and Astrazeneca COVID-19 vaccines, which are both adenovirus vaccines [75,76]. Adenoviral vaccines are yet another vector that can be used to target specific tumor antigens. A barrier to adenovirus-based vaccines are the existing antibodies from previous adenoviral exposures that can neutralize the adenovirus before the vaccine can induce a T cell response. Siemens et al. developed a way to circumvent neutralizing adenoviral antibodies by delivering the adenoviral vaccine targeting PSA (Ad/PSA) with gelfoam, a collagen-based matrix [77]. It was proven to be safe in a phase I trial and the majority of patients demonstrated anti-PSA T cell responses [78]. PSA doubling time were also seen to be increased in about half of the patients. An ongoing phase II trial is studying the use of Ad/PSA in patients with mCRPC (NCT00583024). There is also a phase I study of an adenovirus vaccine with three target antigens; PSA, brachyury, and MUC-1 in patients with mCRPC [79]. Adenovirus vaccines boost high expression levels of the transgenes, but its ability to infect target cells is conditional on the cell’s expression of Ad receptor, which is not universally expressed on cancer cells. 

#### 3.3.3. DNA/mRNA-Based Vaccines

DNA-based cancer vaccines are based on an expression plasmid engineered from a target antigen, which is then presented via MHC class II molecules to activate CD4^+^ T cells. In PCa, prostatic acid phosphatase (PAP) is a promising target antigen because its expression is exclusive to prostatic tissue, both normal and malignant, and has a rodent homolog for preclinical models [80,81]. McNeel et al. administered a DNA vaccine encoding PAP to patients with BCR cancer and observed amplified antigen-specific cytotoxic T-cell responses, termed PAP-specific IFNγ secreting T-cells [81]. They concluded that multiple immunizations were needed to elicit these T-cell immune responses. Some individuals in the study had a ≥200% increase in PSA doubling time and most of these patients had long term PAP-specific IFNγ secreting T-cell immune responses [82].

Another DNA vaccine was assessed in a phase I/II trial for patients with PCa. This vaccine encoded an HLA-A2-binding epitope from PSMA fused to a domain of fragment C of the tetanus toxin. The vaccine was safe and well tolerated, and was shown to induce PSMA-specific CD8^+^ T cells along with a CD4^+^ T cell help. Despite no effect of DNA vaccine dose on outcome, PSADT increased from 11.97 months pre-treatment to 16.82 months over a 72-week follow-up [83].

Messenger RNA (mRNA) vaccines are also in development for immunotherapeutic purposes. mRNA vaccines are created by using bacterial RNA polymerase with the template DNA encoding an antigen of interest. After mRNA translation, the antigen is presented to the immune system to stimulate an immune response. Trials using mRNA vaccines to treat cancer are currently underway for esophageal, pancreatic, gastric, ovarian, and skin cancers [84]. One ongoing trial is studying the use of an mRNA vaccine targeting antigens expressed in prostate cancer complexed with liposomes for stability (RNA-LPX). More specifically, the mRNA encodes for 5 antigens (PAP, PSA and 3 undisclosed antigens) expressed de novo and metastatic prostatic cancer. This mRNA cancer vaccine is being tested with or without adjuvant cemiplimab in men with mCRPC (NCT04382898).

#### 3.3.4. *Listeria monocytogenes*-Based Vaccines

*Listeria monocytogenes* (*Lm*) is an intracellular bacteria pathogen, which has been used as a vaccine vector for the delivery of antigens in various settings [85,86,87]. When antigens are fused to a truncated form of Listeriolysin O (LLO), the fusion protein has been shown to rapidly be taken up by APCs, resulting in the formation of tumor antigen-specific CTLs. In addition to stimulating an anti-tumor response, *Lm*-based immunotherapies have been shown to have immunomodulating effects, reducing the number and function of immunosuppressive Tregs and MDSCs within the TME of mouse models with upregulation of PD-L1 expression [87,88].

ADXS31-142 is a live, attenuated, bioengineered *Lm*-LLO immunotherapy designed to secrete an antigen-adjuvant fusion protein consisting of a fragmented LLO protein fused to human PSA [88]. The agent is currently under exploration in combination with pembrolizumab as part of a phase 1/2 KEYNOTE-046 trial (NCT02325557) and appears to be safe and tolerable in patients with mCRPC. Current results indicate medial overall survival of 33.7 months for patients with mCRPC treated with the combination vaccine and ICI. Improvements in median overall survival have also been seen in patients with prior visceral metastases of 16.4 months compared to 11 months in those undergoing current standard of care [89].

### 3.4. Adenosine Receptor Antagonists

Adenosine receptors A2a and A2b are upregulated by some cancer cells and work to prevent lymphocytes and myeloid cells from infiltrating tumor cells. A2a and A2b are G protein-coupled receptors (GCPRs) that are expressed in PCa cells [90]. Etrumedenant is a new dual adenosine A2a/A2b receptor antagonist developed to target PCa. ARC-6 is an ongoing phase Ib/II open label trial evaluating etrumedenant with zimberelimab (PD-1 inhibitor) and docetaxel in patients with mCRPC (NCT04381832). 

### 3.5. Bi-Specific T-Cell Engagers (BiTEs)

Another developing area in immunotherapeutics for CRPC is bispecific monoclonal antibodies called bi-specific T-cell engagers (BiTEs), which conjugate simultaneously with tumor antigens and T cells. The FDA approved blinatumomab, a BiTE, for use in B-cell acute lymphoblastic leukemia [91]. In a preclinical study, an oncolytic adenovirus was engineered to encode a fibroblast activation protein BiTE to attack stromal fibroblasts in PCa tissue [92]. Pasotuxizumab is a BiTE that engages CD3 on T cells and targets PSMA on PCa cells, and a recent phase I trial showed that it had a dose-dependent PSA response, including two long term responders out of total of nine patients [93]. AMG 160 is a PSMA targeting BiTE being studied in a phase I trial in CRPC patients who are unable to receive or failed taxane therapy (NCT03792841). Preliminary results presented at ESMO Virtual Congress 2020, AMG 160 monotherapy has shown a manageable safety profile, with responses by PSA decline (>50% decline) in greater than one third of patients. In addition, evidence of response via imaging was seen in 2 patients with partial responses with an additional 8 patients demonstrating stable disease. HPN424 is an CD3/PSMA-targeting monoclonal antibody, although it is trispecific rather than bispecific. HPN424 has an albumin-binding domain, which is designed to extend the half-life of the compound. There is a phase I study for HPN424 in patients with mCRPC who have progressed on systemic therapy (NCT03577028).

## 4. Combination Strategies

Due to the complex nature of immunotherapy, multifaceted combination therapies are being investigated. Trials are underway to study various permutations of treatments that include antigen vaccines, DNA vaccines, and checkpoint inhibitors. A current study is examining the effect of a neoantigen DNA vaccine in combination with PROSTVAC, nivolumab and ipilimumab (NCT03532217). Another trial is evaluating ipilimumab in combination with GVAX [94]. Pembrolizumab combination therapies in mCRPC are being investigated in KEYNOTE-365 (NCT02861573) a phase Ib/II study mentioned in the previous section with four different study medications (pembrolizumab, docetaxel, enzalutamide, Olaparib, abiraterone and prednisone). Ongoing studies are investigating combination Sipuleucel-T with atezolizumab (NCT03024216), ipilimumab (NCT01804465), radiation (NCT02463799, NCT01818986, NCT01807065), and chemotherapy (NCT01420965). A list of ongoing studies is included in Table 1.

## 5. Conclusions

Despite the early success of Sipuleucel-T in advanced PCa, immune therapies have proven effective in few patients with mCRPC. The poor response of PCa to these therapies is thought to be multifactorial—likely a result of the complex interplay between the TME, immune evasion mechanisms, and subsequent malignant disease progression. The role of immunotherapy as monotherapy or combination-based therapy in treating metastatic PCa is growing. The incentive for gaining a deeper understanding of the TME and mechanisms of immune resistance lies in the opportunity to further the development of vaccines, checkpoint inhibitors, and combination strategies in the treatment of advanced PCa. 

## Figures and Tables

**Table 1:** Current ongoing clinical trials of immunotherapeutics in prostate cancer.

Agent (s)	Mechanism	Clinical Phase	Indication	Clinical Trial ID
DCVAC/PCa/docetaxel/prednisone	Autologous DC vaccine + chemotherapy	III	mCRPC	NCT02111577 (VIABLE)
Ad/PSA	Adenovirus-based vaccine with PSA gene	II	mCRPC	NCT00583024
W_pro1/cemiplimab	mRNA-based vaccine monotherapy complexed with liposomes +/− IC	I/II	mCRPC	NCT04382898 (PRO-MERIT)
CAR T cell-PSCA (BPX-601)	Autologous T cell-based vaccine	I/II	mCRPC	NCT02744287
CAR T cell-PSMA	CAR T cell-based vaccine	I	mCRPC	NCT01140373
Entrumadenant/zimberelimab/enzalutamide/docetaxel/AB680	Adenosine receptor antagonist + ICI + CD73 inhibitor + ADT + chemotherapy	I/II	mCRPC	NCT04381832 (ARC-6)
AMG160/pembrolizumab	PSMA-targeting Bispecific T-cell Engager + ICI	I	mCRPC	NCT03792841
HPN424	PSMA-targeting Bispecific T-cell Engager	I/II	mCRPC	NCT03577028
PROSTVAC/Ipilimumab/Nivolumab/Neoantigen DNA vaccine	Viral vector-based vaccine + ICI+ DNA vaccine	I	HSPC	NCT03532217
Pembrolizumab/enzalutamide/docetaxel/olaparib/abiraterone/prednisone	ICI + ADT + PARP inhibitor + chemotherapy	I/II	mCRPC	NCT02861573 (KEYNOTE-365)
Atezolizumab/Sipuleucel-T	ICI + DC Vaccine	Ib	mCRPC	NCT03024216
Ipilimumab/Sipuleucel-T	ICI + DC Vaccine	II	mCRPC	NCT01804465
Sipuleucel-T/CT-011/Cyclophosphamide	DC Vaccine + ICI + chemotherapy	I	mCRPC	NCT01420965
Nivolumab/Ipilimumab/Cabazitaxel/Prednisone	ICI + chemotherapy	II	mCRPC	NCT02985957 (CheckMate 650)

mCRPC metastatic castration-resistant prostate cancer; ICI immune checkpoint inhibitor; HSPC hormone-sensitive prostate cancer; PSA prostate-specific antigen; PSMA prostate-specific membrane antigen; DC dendritic cell; ADT androgen deprivation therapy; CAR T chimeric antigen receptor T cells; PARP poly ADP ribose polymerase.

## References

[1] Bahmad H.F., Jalloul M., Azar J., Moubarak M.M., Samad T.A., Mukherji D., Al-Sayegh M., Abou-Kheir W. (2021). Tumor Microenvironment in Prostate Cancer: Toward Identification of Novel Molecular Biomarkers for Diagnosis, Prognosis, and Therapy Development. Front. Genet..

[2] Cheng H.H., Lin D.W., Yu E.Y. (2012). Advanced clinical states in prostate cancer. Urol. Clin. N. Am..

[3] Reimers M.A., Slane K.E., Pachynski R.K. (2019). Immunotherapy in Metastatic Castration-Resistant Prostate Cancer: Past and Future Strategies for Optimization. Curr. Urol. Rep..

[4] Alatrash G., Jakher H., Stafford P.D., Mittendorf E.A. (2013). Cancer immunotherapies, their safety and toxicity. Expert Opin. Drug Saf..

[5] Shiao S.L., Chu G.C., Chung L.W. (2016). Regulation of prostate cancer progression by the tumor microenvironment. Cancer Lett..

[6] Beer T.M., Kwon E.D., Drake C.G., Fizazi K., Logothetis C., Gravis G., Ganju V., Polikoff J., Saad F., Humanski P. (2017). Randomized, Double-Blind, Phase III Trial of Ipilimumab Versus Placebo in Asymptomatic or Minimally Symptomatic Patients with Metastatic Chemotherapy-Naive Castration-Resistant Prostate Cancer. J. Clin. Oncol..

[7] Kwon E.D., Drake C.G., Scher H.I., Fizazi K., Bossi A., van den Eertwegh A.J., Krainer M., Houede N., Santos R., Mahammedi H. (2014). Ipilimumab versus placebo after radiotherapy in patients with metastatic castration-resistant prostate cancer that had progressed after docetaxel chemotherapy (CA184-043): A multicentre, randomised, double-blind, phase 3 trial. Lancet Oncol..

[8] Vitkin N., Nersesian S., Siemens D.R., Koti M. (2019). The Tumor Immune Contexture of Prostate Cancer. Front. Immunol..

[9] Nair S.S., Weil R., Dovey Z., Davis A., Tewari A.K. (2020). The Tumor Microenvironment and Immunotherapy in Prostate and Bladder Cancer. Urol. Clin. N. Am..

[10] Laccetti A.L., Subudhi S.K. (2017). Immunotherapy for metastatic prostate cancer: Immuno-cold or the tip of the iceberg?. Curr. Opin. Urol..

[11] Handa S., Hans B., Goel S., Bashorun H.O., Dovey Z., Tewari A. (2020). Immunotherapy in prostate cancer: Current state and future perspectives. Ther. Adv. Urol..

[12] Madan R.A., Antonarakis E.S., Drake C.G., Fong L., Yu E.Y., McNeel D.G., Lin D.W., Chang N.N., Sheikh N.A., Gulley J.L. (2020). Putting the Pieces Together: Completing the Mechanism of Action Jigsaw for Sipuleucel-T. J. Natl. Cancer Inst..

[13] Wong R.L., Yu E.Y. (2021). Refining Immuno-Oncology Approaches in Metastatic Prostate Cancer: Transcending Current Limitations. Curr. Treat. Options Oncol..

[14] Bander N.H., Yao D., Liu H., Chen Y.T., Steiner M., Zuccaro W., Moy P. (1997). MHC class I and II expression in prostate carcinoma and modulation by interferon-alpha and -gamma. Prostate.

[15] Reits E.A., Hodge J.W., Herberts C.A., Groothuis T.A., Chakraborty M., Wansley E.K., Camphausen K., Luiten R.M., de Ru A.H., Neijssen J. (2006). Radiation modulates the peptide repertoire, enhances MHC class I expression, and induces successful antitumor immunotherapy. J. Exp. Med..

[16] Bryant G., Wang L., Mulholland D.J. (2017). Overcoming Oncogenic Mediated Tumor Immunity in Prostate Cancer. Int. J. Mol. Sci..

[17] Runcie K.D., Dallos M.C. (2021). Prostate Cancer Immunotherapy-Finally in From the Cold?. Curr. Oncol. Rep..

[18] Stultz J., Fong L. (2021). How to turn up the heat on the cold immune microenvironment of metastatic prostate cancer. Prostate Cancer Prostatic Dis..

[19] Sanaei M.J., Salimzadeh L., Bagheri N. (2020). Crosstalk between myeloid-derived suppressor cells and the immune system in prostate cancer: MDSCs and immune system in Prostate cancer. J. Leukoc. Biol..

[20] Fleming V., Hu X., Weber R., Nagibin V., Groth C., Altevogt P., Utikal J., Umansky V. (2018). Targeting Myeloid-Derived Suppressor Cells to Bypass Tumor-Induced Immunosuppression. Front. Immunol..

[21] Kumar V., Patel S., Tcyganov E., Gabrilovich D.I. (2016). The Nature of Myeloid-Derived Suppressor Cells in the Tumor Microenvironment. Trends Immunol..

[22] Lopez-Bujanda Z., Drake C.G. (2017). Myeloid-derived cells in prostate cancer progression: Phenotype and prospective therapies. J. Leukoc. Biol..

[23] Hossain D.M., Pal S.K., Moreira D., Duttagupta P., Zhang Q., Won H., Jones J., D’Apuzzo M., Forman S., Kortylewski M. (2015). TLR9-Targeted STAT3 Silencing Abrogates Immunosuppressive Activity of Myeloid-Derived Suppressor Cells from Prostate Cancer Patients. Clin. Cancer Res..

[24] Idorn M., Køllgaard T., Kongsted P., Sengeløv L., Thor Straten P. (2014). Correlation between frequencies of blood monocytic myeloid-derived suppressor cells, regulatory T cells and negative prognostic markers in patients with castration-resistant metastatic prostate cancer. Cancer Immunol. Immunother..

[25] Muthuswamy R., Corman J.M., Dahl K., Chatta G.S., Kalinski P. (2016). Functional reprogramming of human prostate cancer to promote local attraction of effector CD8(+) T cells. Prostate.

[26] Garcia-Lora A., Algarra I., Garrido F. (2003). MHC class I antigens, immune surveillance, and tumor immune escape. J. Cell Physiol..

[27] Sanda M.G., Restifo N.P., Walsh J.C., Kawakami Y., Nelson W.G., Pardoll D.M., Simons J.W. (1995). Molecular characterization of defective antigen processing in human prostate cancer. J. Natl. Cancer Inst..

[28] Maleki Vareki S. (2018). High and low mutational burden tumors versus immunologically hot and cold tumors and response to immune checkpoint inhibitors. J. Immunother. Cancer.

[29] Berger M.F., Lawrence M.S., Demichelis F., Drier Y., Cibulskis K., Sivachenko A.Y., Sboner A., Esgueva R., Pflueger D., Sougnez C. (2011). The genomic complexity of primary human prostate cancer. Nature.

[30] Theofilopoulos A.N., Baccala R., Beutler B., Kono D.H. (2005). Type I interferons (alpha/beta) in immunity and autoimmunity. Annu. Rev. Immunol..

[31] Lo U.G., Pong R.C., Yang D., Gandee L., Hernandez E., Dang A., Lin C.J., Santoyo J., Ma S., Sonavane R. (2019). IFNγ-Induced IFIT5 Promotes Epithelial-to-Mesenchymal Transition in Prostate Cancer via miRNA Processing. Cancer Res..

[32] Kundu M., Roy A., Pahan K. (2017). Selective neutralization of IL-12 p40 monomer induces death in prostate cancer cells via IL-12-IFN-γ. Proc. Natl. Acad. Sci. USA.

[33] Owen K.L., Gearing L.J., Zanker D.J., Brockwell N.K., Khoo W.H., Roden D.L., Cmero M., Mangiola S., Hong M.K., Spurling A.J. (2020). Prostate cancer cell-intrinsic interferon signaling regulates dormancy and metastatic outgrowth in bone. EMBO Rep..

[34] Vidotto T., Saggioro F.P., Jamaspishvili T., Chesca D.L., Picanço de Albuquerque C.G., Reis R.B., Graham C.H., Berman D.M., Siemens D.R., Squire J.A. (2019). PTEN-deficient prostate cancer is associated with an immunosuppressive tumor microenvironment mediated by increased expression of IDO1 and infiltrating FoxP3+ T regulatory cells. Prostate.

[35] Peng W., Chen J.Q., Liu C., Malu S., Creasy C., Tetzlaff M.T., Xu C., McKenzie J.A., Zhang C., Liang X. (2016). Loss of PTEN Promotes Resistance to T Cell-Mediated Immunotherapy. Cancer Discov..

[36] Chen Y., Sawyers C.L., Scher H.I. (2008). Targeting the androgen receptor pathway in prostate cancer. Curr. Opin. Pharmacol..

[37] Page S.T., Plymate S.R., Bremner W.J., Matsumoto A.M., Hess D.L., Lin D.W., Amory J.K., Nelson P.S., Wu J.D. (2006). Effect of medical castration on CD4^+^ CD25^+^ T cells, CD8+ T cell IFN-gamma expression, and NK cells: A physiological role for testosterone and/or its metabolites. Am. J. Physiol. Endocrinol. Metab..

[38] Shen Y.C., Ghasemzadeh A., Kochel C.M., Nirschl T.R., Francica B.J., Lopez-Bujanda Z.A., Carrera Haro M.A., Tam A., Anders R.A., Selby M.J. (2018). Combining intratumoral Treg depletion with androgen deprivation therapy (ADT): Preclinical activity in the Myc-CaP model. Prostate Cancer Prostatic Dis..

[39] Obradovic A.Z., Dallos M.C., Zahurak M.L., Partin A.W., Schaeffer E.M., Ross A.E., Allaf M.E., Nirschl T.R., Liu D., Chapman C.G. (2020). T-Cell Infiltration and Adaptive Treg Resistance in Response to Androgen Deprivation With or Without Vaccination in Localized Prostate Cancer. Clin. Cancer Res..

[40] Brahmer J.R., Drake C.G., Wollner I., Powderly J.D., Picus J., Sharfman W.H., Stankevich E., Pons A., Salay T.M., McMiller T.L. (2010). Phase I study of single-agent anti-programmed death-1 (MDX-1106) in refractory solid tumors: Safety, clinical activity, pharmacodynamics, and immunologic correlates. J. Clin. Oncol..

[41] Hodi F.S., O’Day S.J., McDermott D.F., Weber R.W., Sosman J.A., Haanen J.B., Gonzalez R., Robert C., Schadendorf D., Hassel J.C. (2010). Improved survival with ipilimumab in patients with metastatic melanoma. N. Engl. J. Med..

[42] Motzer R.J., Escudier B., McDermott D.F., George S., Hammers H.J., Srinivas S., Tykodi S.S., Sosman J.A., Procopio G., Plimack E.R. (2015). Nivolumab versus Everolimus in Advanced Renal-Cell Carcinoma. N. Engl. J. Med..

[43] Calagua C., Russo J., Sun Y., Schaefer R., Lis R., Zhang Z., Mahoney K., Bubley G.J., Loda M., Taplin M.E. (2017). Expression of PD-L1 in Hormone-naïve and Treated Prostate Cancer Patients Receiving Neoadjuvant Abiraterone Acetate plus Prednisone and Leuprolide. Clin. Cancer Res..

[44] Martin A.M., Nirschl T.R., Nirschl C.J., Francica B.J., Kochel C.M., van Bokhoven A., Meeker A.K., Lucia M.S., Anders R.A., DeMarzo A.M. (2015). Paucity of PD-L1 expression in prostate cancer: Innate and adaptive immune resistance. Prostate Cancer Prostatic Dis..

[45] Haffner M.C., Guner G., Taheri D., Netto G.J., Palsgrove D.N., Zheng Q., Guedes L.B., Kim K., Tsai H., Esopi D.M. (2018). Comprehensive Evaluation of Programmed Death-Ligand 1 Expression in Primary and Metastatic Prostate Cancer. Am. J. Pathol..

[46] Bishop J.L., Sio A., Angeles A., Roberts M.E., Azad A.A., Chi K.N., Zoubeidi A. (2015). PD-L1 is highly expressed in Enzalutamide resistant prostate cancer. Oncotarget.

[47] Antonarakis E.S., Piulats J.M., Gross-Goupil M., Goh J., Ojamaa K., Hoimes C.J., Vaishampayan U., Berger R., Sezer A., Alanko T. (2020). Pembrolizumab for Treatment-Refractory Metastatic Castration-Resistant Prostate Cancer: Multicohort, Open-Label Phase II KEYNOTE-199 Study. J. Clin. Oncol..

[48] Hansen A.R., Massard C., Ott P.A., Haas N.B., Lopez J.S., Ejadi S., Wallmark J.M., Keam B., Delord J.P., Aggarwal R. (2018). Pembrolizumab for advanced prostate adenocarcinoma: Findings of the KEYNOTE-028 study. Ann. Oncol..

[49] Topalian S.L., Hodi F.S., Brahmer J.R., Gettinger S.N., Smith D.C., McDermott D.F., Powderly J.D., Carvajal R.D., Sosman J.A., Atkins M.B. (2012). Safety, activity, and immune correlates of anti-PD-1 antibody in cancer. N. Engl. J. Med..

[50] Sharma P., Pachynski R.K., Narayan V., Fléchon A., Gravis G., Galsky M.D., Mahammedi H., Patnaik A., Subudhi S.K., Ciprotti M. (2020). Nivolumab Plus Ipilimumab for Metastatic Castration-Resistant Prostate Cancer: Preliminary Analysis of Patients in the CheckMate 650 Trial. Cancer Cell.

[51] Yu E.Y., Piulats J.M., Gravis G., Laguerre B., Arija J.A.A., Oudard S., Fong P.C.C., Kolinsky M.P., Augustin M., Feyerabend S. (2020). KEYNOTE-365 cohort A updated results: Pembrolizumab (pembro) plus olaparib in docetaxel-pretreated patients (pts) with metastatic castration-resistant prostate cancer (mCRPC). J. Clin. Oncol..

[52] Appleman L.J., Kolinsky M.P., Berry W.R., Retz M., Mourey L., Piulats J.M., Romano E., Gravis G., Gurney H., Bono J.S.D. (2021). KEYNOTE-365 cohort B: Pembrolizumab (pembro) plus docetaxel and prednisone in abiraterone (abi) or enzalutamide (enza)–pretreated patients with metastatic castration-resistant prostate cancer (mCRPC)—New data after an additional 1 year of follow-up. J. Clin. Oncol..

[53] Agarwal N., Vaishampayan U., Green M., di Nucci F., Chang P.Y., Scheffold C., Pal S. (2018). Phase Ib study (COSMIC-021) of cabozantinib in combination with atezolizumab: Results of the dose escalation stage in patients (pts) with treatment-naïve advanced renal cell carcinoma (RCC). Ann. Oncol..

[54] Fizazi K., Drake C.G., Beer T.M., Kwon E.D., Scher H.I., Gerritsen W.R., Bossi A., den Eertwegh A., Krainer M., Houede N. (2020). Final Analysis of the Ipilimumab Versus Placebo Following Radiotherapy Phase III Trial in Postdocetaxel Metastatic Castration-resistant Prostate Cancer Identifies an Excess of Long-term Survivors. Eur. Urol..

[55] Dudzinski S.O., Cameron B.D., Wang J., Rathmell J.C., Giorgio T.D., Kirschner A.N. (2019). Combination immunotherapy and radiotherapy causes an abscopal treatment response in a mouse model of castration resistant prostate cancer. J. Immunother. Cancer.

[56] Philippou Y., Sjoberg H.T., Murphy E., Alyacoubi S., Jones K.I., Gordon-Weeks A.N., Phyu S., Parkes E.E., Gillies McKenna W., Lamb A.D. (2020). Impacts of combining anti-PD-L1 immunotherapy and radiotherapy on the tumour immune microenvironment in a murine prostate cancer model. Br. J. Cancer.

[57] Li F., Glinskii O.V., Mooney B.P., Rittenhouse-Olson K., Pienta K.J., Glinsky V.V. (2017). Cell surface Thomsen-Friedenreich proteome profiling of metastatic prostate cancer cells reveals potential link with cancer stem cell-like phenotype. Oncotarget.

[58] Bansal D., Reimers M.A., Knoche E.M., Pachynski R.K. (2021). Immunotherapy and Immunotherapy Combinations in Metastatic Castration-Resistant Prostate Cancer. Cancers.

[59] Deng Z., Wu Y., Ma W., Zhang S., Zhang Y.Q. (2015). Adoptive T-cell therapy of prostate cancer targeting the cancer stem cell antigen EpCAM. BMC Immunol..

[60] Alzubi J., Dettmer-Monaco V., Kuehle J., Thorausch N., Seidl M., Taromi S., Schamel W., Zeiser R., Abken H., Cathomen T. (2020). PSMA-Directed CAR T Cells Combined with Low-Dose Docetaxel Treatment Induce Tumor Regression in a Prostate Cancer Xenograft Model. Mol. Ther. Oncol..

[61] Kantoff P.W., Higano C.S., Shore N.D., Berger E.R., Small E.J., Penson D.F., Redfern C.H., Ferrari A.C., Dreicer R., Sims R.B. (2010). Sipuleucel-T immunotherapy for castration-resistant prostate cancer. N. Engl. J. Med..

[62] Drake C.G. (2010). Prostate cancer as a model for tumour immunotherapy. Nat. Rev. Immunol..

[63] Flamand V., Sornasse T., Thielemans K., Demanet C., Bakkus M., Bazin H., Tielemans F., Leo O., Urbain J., Moser M. (1994). Murine dendritic cells pulsed in vitro with tumor antigen induce tumor resistance in vivo. Eur. J. Immunol..

[64] Fong L., Carroll P., Weinberg V., Chan S., Lewis J., Corman J., Amling C.L., Stephenson R.A., Simko J., Sheikh N.A. (2014). Activated lymphocyte recruitment into the tumor microenvironment following preoperative sipuleucel-T for localized prostate cancer. J. Natl. Cancer Inst..

[65] Beinart G., Rini B.I., Weinberg V., Small E.J. (2005). Antigen-presenting cells 8015 (Provenge) in patients with androgen-dependent, biochemically relapsed prostate cancer. Clin. Prostate Cancer.

[66] Beer T.M., Bernstein G.T., Corman J.M., Glode L.M., Hall S.J., Poll W.L., Schellhammer P.F., Jones L.A., Xu Y., Kylstra J.W. (2011). Randomized trial of autologous cellular immunotherapy with sipuleucel-T in androgen-dependent prostate cancer. Clin. Cancer Res..

[67] Small E.J., Sacks N., Nemunaitis J., Urba W.J., Dula E., Centeno A.S., Nelson W.G., Ando D., Howard C., Borellini F. (2007). Granulocyte macrophage colony-stimulating factor--secreting allogeneic cellular immunotherapy for hormone-refractory prostate cancer. Clin. Cancer Res..

[68] Higano C.S., Corman J.M., Smith D.C., Centeno A.S., Steidle C.P., Gittleman M., Simons J.W., Sacks N., Aimi J., Small E.J. (2008). Phase 1/2 dose-escalation study of a GM-CSF-secreting, allogeneic, cellular immunotherapy for metastatic hormone-refractory prostate cancer. Cancer.

[69] Podrazil M., Horvath R., Becht E., Rozkova D., Bilkova P., Sochorova K., Hromadkova H., Kayserova J., Vavrova K., Lastovicka J. (2015). Phase I/II clinical trial of dendritic-cell based immunotherapy (DCVAC/PCa) combined with chemotherapy in patients with metastatic, castration-resistant prostate cancer. Oncotarget.

[70] Kongsted P., Borch T.H., Ellebaek E., Iversen T.Z., Andersen R., Met Ö., Hansen M., Lindberg H., Sengeløv L., Svane I.M. (2017). Dendritic cell vaccination in combination with docetaxel for patients with metastatic castration-resistant prostate cancer: A randomized phase II study. Cytotherapy.

[71] Arlen P.M., Gulley J.L., Madan R.A., Hodge J.W., Schlom J. (2007). Preclinical and clinical studies of recombinant poxvirus vaccines for carcinoma therapy. Crit. Rev. Immunol..

[72] Madan R.A., Arlen P.M., Mohebtash M., Hodge J.W., Gulley J.L. (2009). Prostvac-VF: A vector-based vaccine targeting PSA in prostate cancer. Expert Opin. Investig. Drugs.

[73] Kantoff P.W., Schuetz T.J., Blumenstein B.A., Glode L.M., Bilhartz D.L., Wyand M., Manson K., Panicali D.L., Laus R., Schlom J. (2010). Overall survival analysis of a phase II randomized controlled trial of a Poxviral-based PSA-targeted immunotherapy in metastatic castration-resistant prostate cancer. J. Clin. Oncol..

[74] Gulley J.L., Borre M., Vogelzang N.J., Ng S., Agarwal N., Parker C.C., Pook D.W., Rathenborg P., Flaig T.W., Carles J. (2019). Phase III Trial of PROSTVAC in Asymptomatic or Minimally Symptomatic Metastatic Castration-Resistant Prostate Cancer. J. Clin. Oncol..

[75] Sadoff J., Gray G., Vandebosch A., Cárdenas V., Shukarev G., Grinsztejn B., Goepfert P.A., Truyers C., Fennema H., Spiessens B. (2021). Safety and Efficacy of Single-Dose Ad26.COV2.S Vaccine against Covid-19. N. Engl. J. Med..

[76] Emary K.R.W., Golubchik T., Aley P.K., Ariani C.V., Angus B., Bibi S., Blane B., Bonsall D., Cicconi P., Charlton S. (2021). Efficacy of ChAdOx1 nCoV-19 (AZD1222) vaccine against SARS-CoV-2 variant of concern 202012/01 (B.1.1.7): An exploratory analysis of a randomised controlled trial. Lancet.

[77] Siemens D.R., Elzey B.D., Lubaroff D.M., Bohlken C., Jensen R.J., Swanson A.K., Ratliff T.L. (2001). Cutting edge: Restoration of the ability to generate CTL in mice immune to adenovirus by delivery of virus in a collagen-based matrix. J. Immunol..

[78] Lubaroff D.M., Konety B.R., Link B., Gerstbrein J., Madsen T., Shannon M., Howard J., Paisley J., Boeglin D., Ratliff T.L. (2009). Phase I clinical trial of an adenovirus/prostate-specific antigen vaccine for prostate cancer: Safety and immunologic results. Clin. Cancer Res..

[79] Bilusic M., McMahon S., Madan R.A., Karzai F., Tsai Y.T., Donahue R.N., Palena C., Jochems C., Marté J.L., Floudas C. (2021). Phase I study of a multitargeted recombinant Ad5 PSA/MUC-1/brachyury-based immunotherapy vaccine in patients with metastatic castration-resistant prostate cancer (mCRPC). J. Immunother. Cancer.

[80] Terracio L., Rule A., Salvato J., Douglas W.H. (1985). Immunofluorescent localization of an androgen-dependent isoenzyme of prostatic acid phosphatase in rat ventral prostate. Anat. Rec..

[81] McNeel D.G., Dunphy E.J., Davies J.G., Frye T.P., Johnson L.E., Staab M.J., Horvath D.L., Straus J., Alberti D., Marnocha R. (2009). Safety and immunological efficacy of a DNA vaccine encoding prostatic acid phosphatase in patients with stage D0 prostate cancer. J. Clin. Oncol..

[82] Becker J.T., Olson B.M., Johnson L.E., Davies J.G., Dunphy E.J., McNeel D.G. (2010). DNA vaccine encoding prostatic acid phosphatase (PAP) elicits long-term T-cell responses in patients with recurrent prostate cancer. J. Immunother..

[83] Chudley L., McCann K., Mander A., Tjelle T., Campos-Perez J., Godeseth R., Creak A., Dobbyn J., Johnson B., Bass P. (2012). DNA fusion-gene vaccination in patients with prostate cancer induces high-frequency CD8(+) T-cell responses and increases PSA doubling time. Cancer Immunol. Immunother..

[84] Pardi N., Hogan M.J., Porter F.W., Weissman D. (2018). mRNA vaccines—A new era in vaccinology. Nat. Rev. Drug Discov..

[85] Gunn G.R., Zubair A., Peters C., Pan Z.K., Wu T.C., Paterson Y. (2001). Two Listeria monocytogenes vaccine vectors that express different molecular forms of human papilloma virus-16 (HPV-16) E7 induce qualitatively different T cell immunity that correlates with their ability to induce regression of established tumors immortalized by HPV-16. J. Immunol..

[86] Pan Z.K., Ikonomidis G., Pardoll D., Paterson Y. (1995). Regression of established tumors in mice mediated by the oral administration of a recombinant Listeria monocytogenes vaccine. Cancer Res..

[87] Singh R., Dominiecki M.E., Jaffee E.M., Paterson Y. (2005). Fusion to Listeriolysin O and delivery by Listeria monocytogenes enhances the immunogenicity of HER-2/neu and reveals subdominant epitopes in the FVB/N mouse. J. Immunol..

[88] Shahabi V., Reyes-Reyes M., Wallecha A., Rivera S., Paterson Y., Maciag P. (2008). Development of a Listeria monocytogenes based vaccine against prostate cancer. Cancer Immunol. Immunother..

[89] Stein M.N., Fong L., Mega A.E., Lam E.T., Heyburn J.W., Gutierrez A.A., Parsi M., Vangala S., Haas N.B. (2020). KEYNOTE-046 (Part B): Effects of ADXS-PSA in combination with pembrolizumab on survival in metastatic, castration-resistant prostate cancer patients with or without prior exposure to docetaxel. J. Clin. Oncol..

[90] Sek K., Mølck C., Stewart G.D., Kats L., Darcy P.K., Beavis P.A. (2018). Targeting Adenosine Receptor Signaling in Cancer Immunotherapy. Int. J. Mol. Sci.

[91] Huehls A.M., Coupet T.A., Sentman C.L. (2015). Bispecific T-cell engagers for cancer immunotherapy. Immunol. Cell Biol..

[92] Freedman J.D., Duffy M.R., Lei-Rossmann J., Muntzer A., Scott E.M., Hagel J., Campo L., Bryant R.J., Verrill C., Lambert A. (2018). An Oncolytic Virus Expressing a T-cell Engager Simultaneously Targets Cancer and Immunosuppressive Stromal Cells. Cancer Res..

[93] Hummel H.D., Kufer P., Grüllich C., Seggewiss-Bernhardt R., Deschler-Baier B., Chatterjee M., Goebeler M.E., Miller K., de Santis M., Loidl W. (2021). Pasotuxizumab, a BiTE(^®^) immune therapy for castration-resistant prostate cancer: Phase I, dose-escalation study findings. Immunotherapy.

[94] van den Eertwegh A.J., Versluis J., van den Berg H.P., Santegoets S.J., van Moorselaar R.J., van der Sluis T.M., Gall H.E., Harding T.C., Jooss K., Lowy I. (2012). Combined immunotherapy with granulocyte-macrophage colony-stimulating factor-transduced allogeneic prostate cancer cells and ipilimumab in patients with metastatic castration-resistant prostate cancer: A phase 1 dose-escalation trial. Lancet Oncol..

